# Effects of a Risk-Based Licensing Scheme on the Incidence of Alcohol-Related Assault in Queensland, Australia: A Quasi-Experimental Evaluation

**DOI:** 10.3390/ijerph16234637

**Published:** 2019-11-21

**Authors:** Smriti Nepal, Kypros Kypri, John Attia, Tiffany-Jane Evans, Tanya Chikritzhs, Peter Miller

**Affiliations:** 1 School of Medicine and Public Health, University of Newcastle, Callaghan, NSW 2308, Australia; smriti.nepal@uon.edu.au (S.N.); john.attia@newcastle.edu.au (J.A.); 2 Hunter Medical Research Institute, 1 Kookaburra Circuit, New Lambton Heights, NSW 2305, Australia; tiffany.evans@hmri.org.au; 3 National Drug Research Institute, Curtin University, 7 Parker Place, Building 609- Level 2, Technology Park, Bentley, WA 6102, Australia; T.N.Chikritzhs@curtin.edu.au; 4 School of Psychology, Deakin University, Geelong Waterfront Campus, Geelong, VIC 3220, Australia; peter.miller@deakin.edu.au

**Keywords:** alcohol policy, liquor licensing, evaluation, police data, alcohol-related harm, assaults

## Abstract

Amid concerns about increasing alcohol-related violence in licensed premises, Queensland introduced a system of risk-based licensing (RBL) in 2009, the first of five Australian jurisdictions to do so. Under RBL, annual license fees are supposed to reflect the risk of harm associated with the outlet’s trading hours and record of compliance with liquor laws. The objective is to improve service and management practices thereby reducing patron intoxication and related problems. Using police data, we defined cases as assaults that occurred during so-called ‘high-alcohol hours’, and compared a pre-intervention period of 2004–2008 with the post-intervention period 2009–2014. We employed segmented linear regression, adjusting for year and time of assault (high vs. low alcohol hours), to model the incidence of (1) all assaults and (2) a subset that police indicated were related to drinking in licensed premises. We found a small decrease in all assaults (β = −5 per 100,000 persons/year; 95% CI: 2, 9) but no significant change in the incidence of assault attributed to drinking in licensed premises (β = −8; 95% CI: −18, 2). Accordingly, we concluded that the results do not support a hypothesis that RBL is effective in the prevention of harm from licensed premises. There may be value in trialing regulatory schemes with meaningful contingencies for non-compliance, and, in the meantime, implementing demonstrably effective strategies, such as trading hour restrictions, if the aim is to reduce alcohol-related violence.

## 1. Introduction

Violence is common in and around licensed premises in Australia [1]. In the country’s most populous state, 54% of victims and perpetrators involved in assaults in metropolitan areas, and 42% in rural areas had consumed alcohol at licensed premises beforehand [1]. Men and people aged <25 years are at the greatest risk of being involved in such incidents [2]. In 2005, alcohol-related activity in New South Wales (NSW) cost 50 million AUD in police salaries alone [3].

Liquor licensing was introduced almost immediately upon British colonisation of Australia [4] and is now administered independently in each state and territory [5] with the principal objective of preventing intoxication and related harm. In all jurisdictions it is illegal to sell alcohol to an intoxicated person [6], yet intoxication remains common in and around licensed premises. In a study involving covert observation in the entertainment precincts of five cities, 22% to 42% of patrons between 22:00 and 22:59, and 71% to 76% between 02:00 and 02:59 showed signs of intoxication (e.g., slurred speech, lack of coordination, aggression, unsteady on feet), and the proportion of intoxicated individuals increased through the night [7].

Authorities occasionally place special conditions on licensees where they identify significant risk of harm. For example, in 2008, the NSW Liquor Administration Board imposed restrictions on 14 premises in Newcastle in response to police and community complaints about violence and social disorder associated with their operation. New conditions included closing at 03:30 instead of 05:00, and cessation of service at least 30 min before closing [8,9]. The restriction was associated with a one-third reduction in the incidence of assault in the following 18 months and the effect persisted for at least seven years [8,10].

With the goal of reducing alcohol-related harm, the government of Ontario, Canada introduced a province-wide risk-based licensing (RBL) scheme in 2007. In RBL, authorities assess the risk that outlets and applicants pose to the community [11], taking into consideration the type of outlet, its location, occupancy, activities, and trading hours. Applicants are evaluated on their experience, training, and past conduct, including any record of liquor licence infractions. In principle, this was to aid with the allocation of scarce regulatory resources to high risk premises [11].

In January 2009, RBL was initiated in Queensland, Australia [12] and then in the Australian Capital Territory (ACT; 2010), Victoria (2010), NSW (2015), and South Australia (2016) [13]. A similar approach was introduced in New Zealand in 2013 with licensing fees consisting of a base fee (according to venue type) and a risk loading for the number of hours the outlet traded beyond either national default hours or hours set out in local alcohol policies and the outlet’s compliance history in the preceding 18 months [14].

RBL is based on the theory of responsive regulation [15] and was intended to create more nuanced contingencies for good and bad practices than traditional regulations allowed. It applies graded sanctions that ostensibly reflect negative impacts on the community of their operation. The prospect of higher fees is meant to motivate licensees to adopt less risky business models and thereby reduce associated harms. The components taken into consideration vary across jurisdictions and are summarised in Table S1.

In Queensland, RBL requires licensees to pay an annual base fee plus a risk loading that reflects trading hours and compliance history [16]. Higher fees are levied on licensees with premises trading past midnight [17], depending on whether extended hours are one-off or routine [16]. For the compliance history criterion, fees are higher where licensees are served with an infringement notice and penalised or convicted of one or more infringements in the preceding year. Infringements include selling alcohol (1) outside permitted trading hours, (2) to intoxicated patrons, or (3) to minors [17]. Further fees are imposed if disciplinary action is taken because (1) the licensee did not appeal the noncompliance decision, (2) the tribunal confirmed or set aside the decision or substituted another decision, or (3) the licensee was convicted of an offence that contributed to a death or serious assault on or near the premises [16,17]. Fees are indexed annually and payable by 31 July for the 12 months beginning 1 July [16].

To illustrate, we provide the example of a nightclub with an annual base fee of $3757 [18]. If an infringement notice were issued and the licensee paid the fine in the previous licensing year, an additional $6750 compliance history risk loading is charged. If the licensee were convicted of a supply offence and the offence led to a serious assault on or near the premises, an additional compliance history risk loading of $26,980 is charged [18]. If the nightclub has extended trading hours at weekends, risk loadings range from $1045 to $10,430. Similarly, if the nightclub has extended trading hours during the rest of the week, risk loadings range from $1389 to $13,910. In summary, a nightclub trading up to 02:00 on weekend nights, with an infringement in the previous licence period, would be charged a liquor licence fee of $15,727 [18]. Data were not available to put such fees in the context of annual turnover [19].

The Queensland Police and the Office of Liquor and Gaming Regulation control and monitor compliance. While inspections are conducted on high-risk premises, investigations are carried out when the public or police make a complaint against any premises [19]. In 2017, these authorities performed 8785 inspections which prompted 2615 investigations. However, given that there were more than 6000 on-license premises across the state [20], each presumably trading for several thousand hours per year, the probability of being charged even for common offences, such as serving intoxicated patrons, is miniscule. Indeed, there were only 66 successful prosecutions in 2017 [19].

In their evaluation of RBL in the ACT, Mathews and Legrand [21] found that while offences in night-time entertainment precincts subject to RBL declined by 25%, all offences in the ACT declined by 21% over the same period (May 2010 to December 2012), such that the change could not be attributed to RBL. In a recent study, Curtis et al. [22] (including some members of the present research team) examined emergency department data in Victoria and Queensland, finding no association between the introduced RBL and change in the incidence of injury presentations in either state [22]. Our aim in this study was to examine whether the introduction of RBL in Queensland was associated with change in the incidence of assault recorded by police across the state [22].

## 2. Methods

### 2.1. Design

We employed a quasi-experimental design with pre- and post-RBL observations across the entire state of Queensland. We investigated the feasibility of employing within- and between-state control data to account for possible changes in other determinants of assault, but, for reasons explained below, were unable to identify suitable comparators.

We specified the pre-RBL period as 1 January 2004 to 31 December 2008, and the post-intervention period as 1 January 2009 to 31 December 2014. Given that RBL aims to reduce harm by modifying the practices of licensees, we also estimated the association with the subgroup of assaults judged by police to have occurred at or near licensed premises.

### 2.2. Outcome

We utilised the *International Guide for Monitoring Alcohol Consumption and Related Harm (MACRH)* guidelines [23] to select an indicator of alcohol-related assault. Applying the first recommendation, we identified cases in which police judged the offender to have been affected by alcohol. In a previous study, we examined the validity of this indicator, finding that the extent and pattern of missing data on alcohol involvement made it highly unreliable [24]. Accordingly, we applied the *MACRH* second recommendation, defining cases as assaults occurring at times that they are most likely to be alcohol-related, so-called “high alcohol hours” (HAH) (Table S2) [25]. Cases included offenders of any gender or age in the following crime categories: grievous bodily harm, wounding, driving causing grievous bodily harm, serious assault, assault causing bodily harm, aggravated assault, common assault, and minor assault.

HAH for all Australian jurisdictions were established in previous research using blood alcohol concentration (BAC) records of drivers involved in traffic injury crashes from 1990 to 1997 [25]. In that research, data were aggregated by day of the week and the time-of-day when injury occurred, creating six periods of four consecutive hours, producing a mutually exclusive, exhaustive set of 42 periods. For each period the proportion of BACs exceeding the legal limit for driving (0.05 g/dL) was calculated. The researchers estimated the mean proportion of BACs exceeding the limit and the standard deviation for each category. Where the proportion of BACs exceeding the limit was ≥1 standard deviation above the overall mean in a period, they classified it as HAH [25].

While noting that alcohol involvement examined in the context of roadside testing of drink-drivers may not generalise to the incidence of alcohol-related assault, we considered the benefit of identifying cases on the basis of an objective measure, namely a breath or blood alcohol test, to outweigh that risk. Notably, the hours specified as HAH via this method were similar to those used in other studies [7,26], however, it is not clear on what basis the authors of those studies selected the times.

### 2.3. Statistical Analyses

Using census-based estimates, we calculated the incidence of assault per 100,000 population per year [27]. Examining the Breusch–Godfrey test and Durbin–Watson statistics, we found no evidence of autocorrelation. To determine whether the implementation of RBL was followed by a lower incidence of assault, we employed segmented linear regression [28], modelling the crude incidence pre- and post-RBL, allowing these slopes to differ in the two periods.

We modelled segments for the pre- and post-RBL periods using: (1) a continuous variable for time that estimated the mean change in incidence pre RBL, (2) an indicator variable for pre vs. post periods estimating the level of change in the number of assaults post RBL, and (3) a variable for time post RBL estimating mean change in the post-RBL trend compared with pre RBL. We used Stata/IC 14 to conduct the analysis.

### 2.4. Sensitivity Analyses

In a sensitivity analysis, we excluded assaults occurring in 2008 on the basis that licensees were likely to have known in advance that RBL would be introduced and may have changed their business practices before it came into effect. In a second sensitivity analysis, we excluded assaults occurring in 2009 because the element of RBL with the most scope to influence behaviour, i.e., the compliance history risk loading, could not be used to determine licensing fees in the first year. Accordingly, we hypothesised that if the prospect of a fee increase was critical to RBL’s effectiveness, change might not be evident until after 2009. Both of these analyses addressed the possibility that our primary analysis underestimated the effect due to blurring of the pre-post contrast.

### 2.5. Supplementary Analyses

In order to account for other determinants of assault, we originally planned to include within-state and between-state comparisons. Employing the third recommendation from *MACRH* [23], to control for service delivery variables, we utilised events from the same dataset that were least likely to be alcohol-related, i.e., ‘low alcohol hours’ (LAH) [29]. We hypothesised that if RBL were effective, we would see a greater decrease in assaults during HAH than LAH. For the comparison, we produced an HAH/LAH ratio, and tested whether post-RBL associations differed in HAH and LAH assaults. We used terms to represent the interaction between this variable and (a) the pre/post indicator (category x period) and (b) time post-intervention (category × time post). However, contrary to our expectation, the number of assaults per hour was higher during LAH than during HAH, so we decided that this would not be an appropriate comparison and excluded it from the primary analysis. For the purpose of transparency, we present the findings as supplementary materials in Figure S1 and Table S3.

Similarly, for a between-state comparison, we identified Western Australia (WA) as the most similar jurisdiction to Queensland, in which RBL had not been introduced during the post-RBL period in Queensland. However, on examining the data, we found that WA had a markedly higher incidence of assault to begin with and, more importantly, a clearly different trend in the years 2004–2008, before the introduction of RBL in Queensland. Accordingly, we did not rely on that analysis but provide it as supplementary materials in Figure S2 and Table S4.

## 3. Results

**Figure 1:**
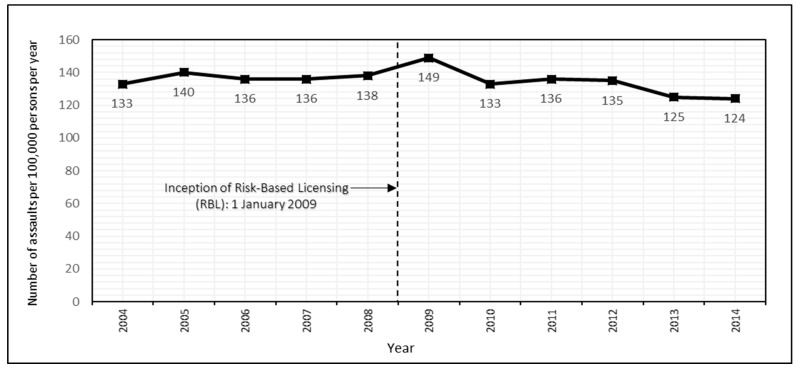
Crude annual assault incidence rates during “high alcohol hours” in Queensland.

From 2004 to 2014, Queensland Police recorded 242,107 assaults of which 62,703 occurred during HAH. Figure 1 presents annual crude incidence rates of HAH assault from 2004 to 2014, showing that rates neither increased nor decreased markedly in the pre and post periods.

Table 1 presents model parameters from the segmented linear regression analysis and shows no significant step change in the incidence of HAH assaults immediately after RBL was introduced. The slope change estimates showed a reduction of five cases per year in all HAH assaults following RBL (β = −5.32, 95% CI: −1.59, −9.04). That decrease was not sensitive to the exclusion of 2008 (β = −5.50, 95% CI: −0.88, −10.1) or 2009 (β = −3.82, 95% CI: −0.35, −7.30) from the analysis (Tables S5 and S6).

**Figure 2:**
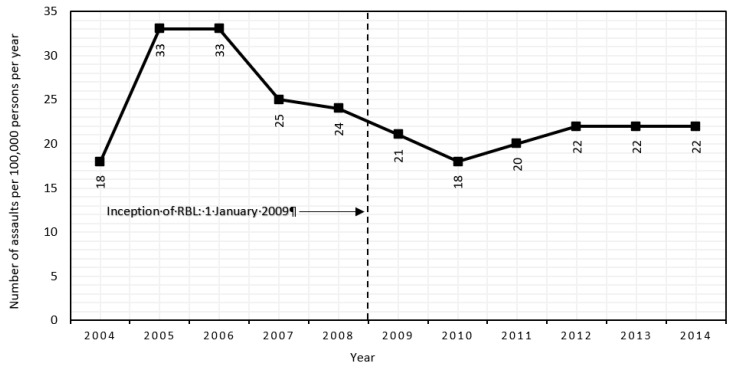
Crude annual incidence rates of assault during “high alcohol hours” related to licensed premises.

Given that RBL aimed to reduce harm by modifying licensee behaviour, we assessed the association in relation to assaults that police had attributed to licensed premises. There were 24,700 such events from 2004 to 2014, of which 11,012 occurred during HAH. Figure 2 presents the crude annual incidence rates of this outcome, showing a peak in 2005–2006, well before the inception of RBL.

The regression model showed neither a significant step change nor a slope change in HAH assaults related to licensed premises (Table 2). Additionally, nothing in the within- or between-state comparisons (Tables S3 and S4) suggested that RBL was effective in Queensland.

## 4. Discussion

Following the implementation of RBL, we found a small decrease in the incidence of total assaults during HAH, but no association between RBL and HAH assaults that police determined were related to licensed premises. None of the supplementary within- or between-state comparisons suggested that RBL was effective in reducing alcohol-related harm in Queensland.

A strength of this study is that the multiple pre- and post-intervention observations allowed for adjustment for secular trends. A common approach in time series analysis is to increase the sample size by disaggregating numerators into monthly or quarterly counts, however, this requires additional parameters to adjust for seasonal or month-to-month fluctuation. The method we employed, by eliminating the need to account for seasonality, was simpler and more efficient, obviating the need for parameters whose net effect is decreased statistical power [30]. Furthermore, the analytic choice best reflected the nature of our research question, which concerned the effect of an intervention rather than the trends per se.

Despite extensive efforts, we were unable to identify a suitable counterfactual condition and therefore could not control for history bias [31], i.e., the possibility that unidentified or insufficiently adjusted factors over- or under-estimated the effect of RBL. While advantageous for reasons explained above, using annual rather than monthly or quarterly data made it impossible to adjust models for a major contemporaneous determinant of drinking, namely, the alcopops tax, which came into effect in April 2008 [32]. Studies of national sales data suggest that this tax increase substantially reduced the consumption of the pre-mixed spirit drinks it targeted and also the overall consumption of alcohol, by around 2% nationally, effects that appear to have been sustained [33].

Assuming that the alcopops tax effect was similar in Queensland, we risked a Type I error by misattributing reductions in assault caused by lower alcohol consumption to the effects of RBL. The lack of a significant reduction specifically in assaults attributable to licensed premises, where RBL would be expected to exert its hypothesised effects, therefore makes an inference of no effect more secure. 

Crime surveys suggest that approximately half of assaults go unreported [34], such that the incidence rates we reported will be underestimates. In addition, the unavailability of premise-level data precludes the investigation of whether RBL was associated with changes in the behaviour of licensees. 

We acknowledge that it was not ideal to rely on an indicator in which the specification of HAH came from the analysis of traffic crash data. However, we reasoned that being pegged to an objective measure (namely, blood alcohol level) that is highly correlated with impairment was advantageous. There would be value in establishing the prognostic value of blood alcohol level specifically in relation to assaults that attract police attention.

Our findings align with those from the ACT [21] but there are noteworthy differences between the studies, principally our use of a temporal surrogate informed by analysis of the police data and longer study periods. As occurred in the ACT study [21], there may be value in interviewing regulators, police, and licensees to understand why RBL seems not to have been effective in Queensland. Curtis et al. [22] found no association between RBL and injury presentations, but they included only two years of pre-RBL data for Queensland, thus having relatively low statistical power to estimate the association of interest. Building on two relatively weak tests of the hypothesis, the present study adds weight to the inference that RBL is at least not highly effective in reducing the incidence of assault.

RBL aims to modify licensee behaviour; however, the supposed mechanism of effect appears at odds with the basic principles of behaviour modification. The observations underpinning operant conditioning are that behaviour followed by favourable stimuli (reinforcement) tends to persist, while behaviour followed by aversive stimuli (punishment) tends to diminish [35]. In the operant conditioning of alcohol outlet operators, favourable stimuli include profit from the sale of alcohol to intoxicated patrons. RBL might benefit from exposing low risk licensees to favourable stimuli [35], for example, regulators could pool the fines for infringements collected from licensees and reward the operators of low-risk outlets for their compliance.

Trials in various organisms, including humans, show that for a punishment contingency (e.g., a fine for serving an intoxicated patron) to be effective, it should be sufficiently aversive that the target tries hard to avoid it. It should also be administered soon after the breach to avoid weakening the contingency [35]. By employing a stepped approach to sanctions, RBL delays delivery of the aversive outcome, increasing the propensity of licensees to habituate to the conditions and continue offending. In Australia, liquor licence breaches frequently go unpunished or are punished so ineffectually [22] that the regimes become objects of ridicule and the higher fees are merely a “cost of doing business”. In other words, breaches are too often undetected and punishment is insufficiently aversive to bring operator self-interest into line with the public interest of a safe environment.

A decade ago, as research accumulated on the effects of countermeasures to violence from licensed premises [36,37,38], Stockwell argued that it was time to move from a “one bar at a time” approach to regulating the whole market “one hour at a time” [39]. The idea aligns with theory [40] and research across the spectrum of population health activity, from infectious disease to tobacco control [41]. Since then, evidence for the effectiveness of such whole-of-market policies has strengthened (e.g., [42,43]), while greater scrutiny of targeted approaches reveals them to be largely ineffective (e.g., [44]).

## 5. Conclusions

We found that RBL was not associated with a marked decrease in the incidence of assault that was specific to licensed premises. Accordingly, the results do not support the hypothesis that RBL is effective in the prevention of harm from licensed premises. There may be value in devising and trialing regulatory approaches with meaningful contingencies for compliance and non-compliance, but such policies are unlikely to be acceptable to licensees. In the meantime, if reducing late night alcohol-related violence is the goal, governments should consider approaches shown to be most effective in reducing alcohol-related violence, namely, earlier cessation of alcohol sales [43].

## Figures and Tables

**Table 1:** Change in the incidence of assault during “high alcohol hours” from pre- to post-RBL implementation in Queensland.

Change in Assault	β Coefficient (Number of Assaults per 100,000 Persons per Year)	95% Confidence Interval	*p*-Value
Change pre-RBL	0.97	−1.38, 3.33	0.36
Step change	10.6	−3.78, 24.9	0.13
Slope change	−5.32	−1.59, −9.04	0.01

**Table 2:** Change in incidence of assault during “high alcohol hours” related to licensed premises, from pre to post RBL in Queensland, Australia.

Change in Assault	β Coefficient (Number of Assaults per 100,000 Persons per year)	95% Confidence Interval	*p*-Value
Change pre-RBL	0.45	−5.01, 5.90	0.85
Step change	−8.21	−18.4, 2.01	0.10
Slope change	0.09	−5.45, 5.64	0.97

## Supplementary Materials

The following are available online at https://www.mdpi.com/1660-4601/16/23/4637/s1, Figure S1. Crude annual assault during ‘high alcohol hours’ (HAH) and ‘low alcohol hours’ (LAH), Queensland. Figure S2. Crude annual assault during ‘high alcohol hours’ (HAH) and ‘low alcohol hours’ (LAH), Western Australia. Table S1: Risk-based licensing in Australian jurisdictions. Table S2. High alcohol hours and low alcohol hours for Queensland, Australia. Table S3. Comparison between assaults during high alcohol hours (HAH) and low alcohol hours (LAH) (Within-state comparison). Table S4. Comparison between assaults during ‘high alcohol hours’ and ‘low alcohol hours’ in Queensland and Western Australia. Table S5. Difference in assaults during high alcohol hours (HAH) from pre- to post-RBL in Queensland, Australia (excluding 2008). Table S6. Difference in assaults during high alcohol hours (HAH) from pre- to post-RBL in Queensland, Australia (excluding 2009).
