# Supplementary material for: Effects of a Risk-Based Licensing Scheme on the Incidence of Alcohol-Related Assault in Queensland, Australia: A Quasi-Experimental Evaluation
Source: Int J Environ Res Public Health. 2019 Nov 21;16(23):4637. doi: 10.3390/ijerph16234637 (PMC6926782; doi:10.3390/ijerph16234637)
Supplement: Supplementary file 1 [file ijerph-16-04637-s001.pdf]

Table S1: Risk-based licensing in Australian jurisdictions

Jurisdiction	Base fee	Trading hours	Compliance history	Venue capacity	Location
ACT	✓	✓		✓	
NSW	✓	✓	✓	✓	✓[1]
Queensland	✓	✓	✓		
SA	✓	✓		✓	
Victoria	✓	✓	✓	✓[2]	

[1] Applicable when compliance history risk loading is applied and venues are located in the Kings Cross and Sydney Central Business District.

[2] Applicable when compliance history risk loading is applied.

Table S2. High alcohol hours and low alcohol hours for Queensland*, Australia

**High alcohol hours**	**Low alcohol hours**
Sunday 18:00 - Monday 06:00	Monday 06:00 - Monday 14:00
Tuesday 22:00 - Wednesday 02:00	Tuesday 10:00 - Tuesday 14:00
Wednesday 22:00 - Thursday 02:00	Wednesday 06:00 - Wednesday 14:00
Thursday 18:00 - Friday 02:00	Thursday 06:00 - Thursday 14:00
Friday 22:00 - Saturday 06:00	Friday 06:00 - Friday 10:00
Saturday 18:00 - Sunday 06:00	-----------

***Based on the National Alcohol Indicators Project [1]

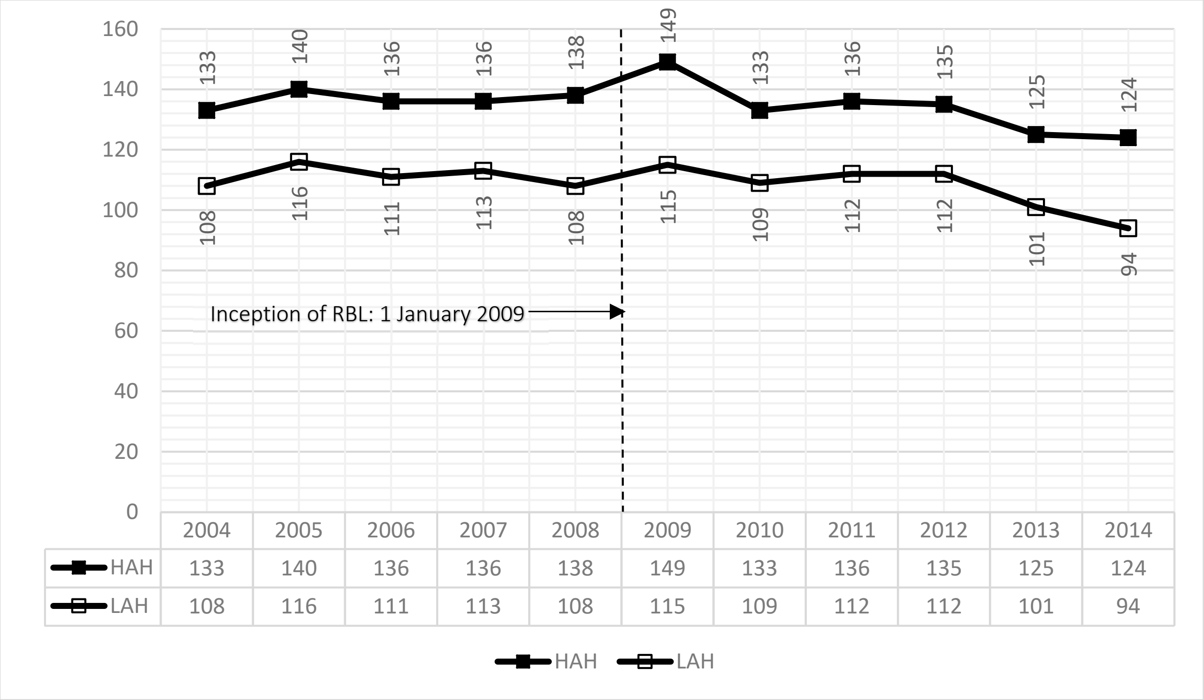

Figure S1. Crude annual assault during ‘high alcohol hours’ (HAH) and ‘low alcohol hours’ (LAH), Queensland

Table S3. Comparison between assaults during high alcohol hours (HAH) and low alcohol hours (LAH) (Within-state comparison)

Change in assault	β coefficient (number of assaults per year)	95% Confidence Interval	p-value
Reference: LAH	24.9	20.4 to 29.6	<0.001
Change in HAH and LAH pre-RBL	0.16	-1.62 to 1.93	0.853
Step change in LAH	7.99	-0.05 to 16.0	0.051
Step change in HAH	11.4	-1.27 to 24.2	0.074
Difference in step change	3.45	-11.2 to 18.1	0.624
Slope change in LAH	-3.84	-6.37 to -1.31	0.006
Slope change in HAH	-4.40	-7.48 to -1.32	0.008
Difference in slope change	-0.55	-3.64 to 2.55	0.711

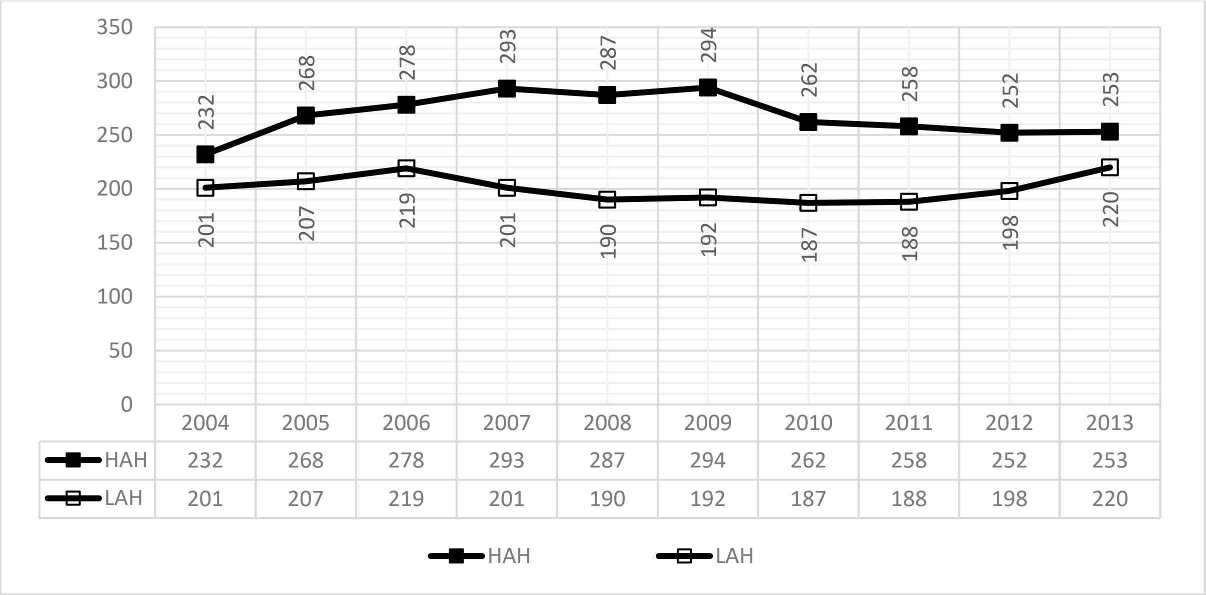

Figure S2. Crude annual assault during 'high alcohol hours' (HAH) and 'low alcohol hours' (LAH), Western Australia

Table S4. Comparison between assaults during 'high alcohol hours' and 'low alcohol hours' in Queensland and Western Australia

Change in assault	β Coefficient (number of assaults per year)	95% Confidence Interval	p-value
Average difference Queensland vs WA pre-RBL	-103	-119 to -87.8	<0.001
Average difference HAH vs LAH pre-RBL	28.6	12.9 to 44.3	0.001
Change in HAH and LAH pre-RBL period, in WA	-4.03	-10.0 to 1.94	0.177
Difference, between the states, in HAH and LAH in pre-RBL	-19.3	-25.7 to -13.0	<0.001
Step change in LAH	-20.1	-45.4 to 5.24	0.115
Difference, between the states, in step change	-6.10	-53.0 to 40.8	0.791
Slope change in LAH	10.8	1.67 to 19.9	0.022
Difference, between the states, in slope change	33.3	20.4 to 46.3	<0.001

Table S5. Difference in assaults during high alcohol hours (HAH) from pre- to post-RBL in Queensland, Australia (excluding 2008)

Change in assault	**β coefficient (number of assaults per year)**	**95% Confidence Interval**	**p-value**
Change in HAH in pre-RBL	1.15	-2.30, 4.60	0.445
Step change in HAH	10.0	-6.36, 26.4	0.185
Slope change in HAH	-5.50	-0.877, -10.1	0.027

Table S6. Difference in assaults during high alcohol hours (HAH) from pre- to post-RBL in Queensland, Australia (excluding 2009)

Change in assault	**β coefficient (number of assaults per year)**	**95% Confidence Interval**	**p-value**
Change in HAH in pre-RBL	0.97	-1.54, 3.49	0.379
Step change in HAH	3.59	-8.54, 15.7	0.479
Slope change in HAH	-3.82	-0.347, -7.30	0.036

References

1. Chikritzhs T, Stockwell T, Heale P, Dietze P, Webb M. National alcohol indicators project technical report no. 2: Trends in alcohol-related road Injury in Australia, 1990-1997. Perth: National Drug Research Institute, Turning Point, Alcohol and Drug Centre Inc.; 2000.
